# The Relationship between Perceived Infectability and Psychological Well-being: The Mediating Role of Covid-19 Anxiety

**DOI:** 10.11621/pir.2023.0205

**Published:** 2023-06-30

**Authors:** S. Mushkbar Fatima, Saira Khan, Rayna Sadia

**Affiliations:** a National Institute of Psychology, Quaid-i-Azam University, Islamabad, Pakistan; b Riphah International University, Gulberg Greens Campus, Islamabad, Pakistan; c Rawalpindi Women University, Rawalpindi, Pakistan

**Keywords:** perceived infectability, coronavirus anxiety, psychological well-being, mediation, COVID-19

## Abstract

**Background:**

COVID-19 has adversely affected economies and individuals globally. To this day, countries are facing the economic effects of the pandemic directly, and individuals’ mental health is in danger as they are still indirectly dealing with the pandemic. It is imperative to understand how pandemic-related anxiety affected individuals’ mental health so that all stakeholders can take essential remedial steps.

**Objective:**

The current research aimed to investigate the relationship between Perceived Infectability, Coronavirus Anxiety, and Psychological Well-being. It also sought to explore the role of coronavirus anxiety in mediating between perceived infectability and psychological well-being.

**Design:**

A cross-sectional correlational study design and non-probability convenience sampling technique were used to collect the data. The data were collected from 321 Pakistani adults, who filled out Google forms on the Perceived Infectability subscale of the Perceived Vulnerability to Disease Scale; the Coronavirus Anxiety Scale; and the Psychological Well-being Scale.

**Results:**

Correlation analysis indicated that both perceived infectability and coronavirus anxiety were negatively related to psychological well-being. However, a significant positive relationship was observed between perceived infectability and coronavirus anxiety. Our findings further proved the mediating role of coronavirus anxiety between perceived infectability and the psychological well-being of adults.

**Conclusion:**

Understanding perceived infectability and its association with COVID-19 anxiety and psychological well-being is pertinent in this post-pandemic period in both developing and developed nations. The post-pandemic world is still being jolted with the aftereffects of the pandemic. An in-depth understanding of how individuals Coped with the pandemic, might help in designing better intervention and community health programs after the pandemic, and it could also help in preparing for the crises attending future pandemics (if any).

## Introduction

In December 2019, a pneumonic outbreak in Wuhan, China turned into a global pandemic. This outbreak, later termed Coronavirus (COVID-19), caused considerable concern, fear, and discomfort in healthcare settings globally in terms of potential contamination (Morawska et al., 2020). Pakistan, being a neighbor of the epicenter of COVID-19, got its first case of the virus on February 26,2020 in Karachi (Naqvi et al., 2020); afterwards, the virus spread across the country rapidly. This rapid spread led to smart lockdowns and closures of all public institutions (including educational ones) to control the spread of the virus.

Due to its contagious nature and high transmission rate, COVID-19 spread worldwide. With the surge in cases, individuals’ concerns about the risk of getting the disease and perceived infectability increased (Van Bavel et al., 2020). Developing countries like Pakistan were at a higher risk due to lack of awareness and limited resources. The virus could affect both the immune-suppressed and normal populations. The risk of contracting the virus led to perceived infectability, which further resulted in inducing coronavirus anxiety (Ovetuniji et al., 2020). In these uncertain times, constant feelings of risk and worry about contracting the virus had an unprecedented and negative impact on well-being. Both the normal and vulnerable populations were at high risk of COVID-19 related stressors (anxiety) as the situation got more muddled and severe day by day, with no clear end in sight and with the nonavailability of the vaccine for the general public and underprivileged.

In Pakistan, the third wave of CO VID-19 (May 2020, the time of the present research) and the lengthy process of providing the vaccines contributed greatly to anxiety. People were surrounded by a great deal of uncertainty and fear because they had faced a new, unusual, and unfamiliar health-related threat with limited and continuously evolving information. An individuals susceptibility to anxiety and vulnerability increases in any crisis, and they actively engage in information-seeking from different sources to reduce their worries. However, the media coverage of the recent pandemic amplified the global fear and caused significant psychological distress among individuals (Bendau et al., 2020) as in the case of other pandemics (Bernstein et al., 2019) and related events (Garfin et al., 2015). These factors directly affected peoples well-being (Amin, 2020), and are therefore a great concern for the scientific community.

During past pandemics, investigations have been directed at the effect of risk perception of the disease on the sentiment of anxiety (Bults et al., 2011), and how mass tragedies, especially those including uncontrolled illnesses, regularly trigger anxiety; such anxiety is known to create disturbances in behavior (Balaratnasingam & Janca, 2006). Since previous studies have established that pandemic-related anxiety dramatically affects individuals’ mental health, it is imperative to understand how COVID-19 related anxiety affects the public’s psychological well-being (Wang et al., 2021).

Coronavirus anxiety and perceived infectability are real threats to people’s psychological well-being, since up until now, people have drawn an unmistakable and hopeless picture of what the present and future holds for a great part of the world as it wrestles with the coronavirus (Arden, 2020); the fear of transmission and life-­threatening nature of the virus is affecting the psychological well-being of populations overall (Feinman et al., 2020). In Pakistan, very few studies until now have addressed the psychological aspects of the virus during the coronavirus era. This means that these aspects need more exploration, since much of our attention is being taken up by the danger of this disease, which is greatly disturbing peoples mental health (Mukhtar, 2020). Moreover, all the prevention programs and strategies imposed by the Pakistani government primarily focused on the physical aspect of the virus and did not provide any information about the psychological aspects of the current pandemic. Yet, the psychological fallout is a very important area of concern not only around the world but also for developing countries like Pakistan, where the mental health of the population is already overburdened.

Therefore, the current study aimed to investigate the relationship between perceived infectability, coronavirus anxiety, and psychological well-being. The present study further investigates the mediating role of coronavirus anxiety within the Pakistani population due to the current global pandemic and will add to knowledge about the psychological aspects of the coronavirus outbreak in Pakistan.

## Methods

### Participants

A cross-sectional correlational study design and non-probability convenience sampling technique were used to collect the data. Prior to data collection, formal approval of the research design was obtained from the research ethics committee (Psy/IRB/Letter-AOO 12), followed by receiving permission from the original authors of the measures used in the present study. The questionnaires were constructed electronically via Google forms and distributed through emails and other communication apps by creating a link where respondents had to click to get access to the questionnaire.

The consent form for the participation included all the necessary information about the researchers and the purpose of the research, accompanied by contact information for research-related queries and assurance about the anonymity and confidentiality of the data. Participants accessed the questionnaire after providing their consent. Instructions were provided before the participants filled out the questionnaire. Lastly, each questionnaire concluded with a note of appreciation to the participant for their participation. The age range of the sample ***(N=*** 321), which had a response rate of 64%, ranged from ranged from 18 to 65 years ***(M*** = 28.88, ***SD=*** 10.46). The sample was comprised of 60.1% female and 39.9% male participants. Among them, 16.8% were older adults, and 83.2% were younger. Moreover, 74.8% had intermediate or bachelor’s degrees, while 25.2% had master’s degrees and a higher level of education.

### Questionnaires

#### The Perceived Vulnerability to Disease Scale

 This instrument, developed by Duncan and colleagues (2009), is used to assess risk perception. The scale has 15 items with two subscales; Perceived Infectability (7 items) evaluates the individual’s convietion about his helplessness in the face of an overwhelming illness, and Germ Aversion (8 items) assesses the individuals level of uneasiness in settings that portend a particularly high potential for microorganism transmission. The participants respond on a 7-point Likert Scale (1-7) from Strongly Disagree to Strongly Agree, with a few reverse coded items (3, 5, 11, 1, 13, & 14). The Cronbachs Alpha reliability of both scales and overall composite scale ranged between .74 to .87 (Duncan et al., 2009). For the present research only the Perceived Infectability subscale was used; high scores on the scale represented high fear of infectability by the Coronavirus, with a score range of 7-49.

#### The Psychological Well-being Scale

 This 18-item scale, developed by Ryff and Keyes (1995), assesses individuals’ psychological well-being on a 7-point Likert scale (1 = Strongly Agree to 7 = Strongly Disagree). Overall, the scale has satisfactory reliability (a = .82) and scores ranged between 18 to 126. High scores represent higher well-being since positive statements are reverse coded (items: 1,2, 3, 8, 9, 11,12, 13, 17, & 18).

#### The Coronavirus Anxiety Scale

 This 5-item scale, developed by Lee (2020), was used to evaluate the COVID-related anxiety of the study participants. Participants responded on a 5-point Likert scale, which ranged from 0 (not at all) to 4 (nearly every day) about their anxiety during the previous 14 days, with high scores (20) representing higher anxiety and low scores (0) lower anxiety. The scale had satisfactory reliability (a= .93).

### Hypotheses of the Study

Based on the literature and aims of the study, the following hypotheses were formulated.

1. There will be a positive association between perceived infectability and coronavirus anxiety among adults.
2. Coronavirus-related anxiety will be negatively associated with the psychological well-being of adults.
3. Coronavirus-related anxiety will mediate the association between perceived infectability and the psychological well-being of adults.

### Procedure

Cronbachs Alpha reliability and correlational analysis were computed using SPSS. Similarly, mediation analysis was carried out on Andrew Hayes’ SPSS Process Macro through model 4 without any control variables. Alpha reliabilities were in the acceptable to good range.

## Results

*Table 1* indicates that a significant positive relationship between perceived infectability and corona virus anxiety exists. Similarly, a significant negative relationship was apparent between perceived infectability and coronavirus anxiety, and psychological well-being.

**Table 1:** Correlation between Perceived Infectability, Coronavirus Anxiety, & Psychological Well-Being among Adults (N = 321)

Variables	α	*M*	*SD*	2	3
1 Perceived infectability	.76	*29.67*	7.57	.47**	-.37**
2 Coronavirus anxiety	.86	4.24	4.06	-	-.50**
3 Psychological well being	.79	81.62	14.79	-	-

Note. **p < .01.

*Table 2* confirms the mediating role of coronavirus anxiety in the relationship between perceived infectability and psychological well-being. As evident, the model without a mediator accounted for a 13 % variance in psychological well-being due to perceived infectability, whereas in model 2, both perceived infectability and coronavirus anxiety significantly and negatively predicted psychological well-being, accounting for a 17 % variance.

**Table 2:** The mediating Role of Coronavirus Anxiety in the Relationship between Perceived Infectibality & Psychological Well-Being among Adults (N = 321)

Model	B	SE	P	CI (lower)	CI (Upper)
Model without a Mediator					
Constant	102.79	3.12	.00	96.66	108.92
PI—PWB(c)	-.71	.10	.00	-.91	-.51
*R^2^* (Y X)	.13				
Models with a Mediator					
Model 1: Coronavirus Anxiety as a Dependent Variable					
Constant	-3.31	.81	.000	-4.90	-1.72
PI------CA (a)	.25	.03	.000	.20	.31
*R^2^*	.23				
Model 2: Psychological Well–Being as a Dependent Variable					
Constant	97.70	2.94	.000	91.92	103.47
CA----PWB(b)	-1.54	.20	.000	-1.93	-1.15
PI----PWB (c’)	-.32	.11	.002	-.53	-.11
Indirect effect	-.39	.004		-.04	-.02
R^2^(Y, M, X)	.27				

*Note, (sobel z = -6.03. p < .01). PI = Perceived Infectability; CA = Coronavirus Anxiety; PWB = Psychological Well-Being*.

**Figure 1.**
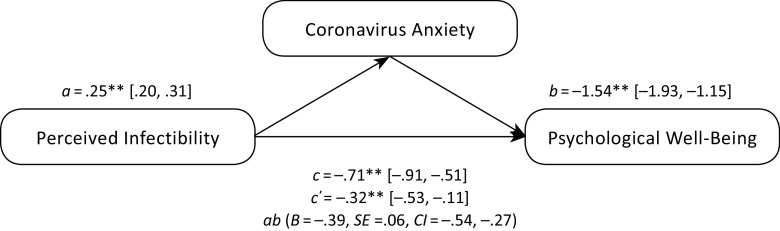
The mediating role of coronavirus anxiety in the relationship between perceived infectability and psychological well-being

## Discussion

One of the main objectives of the present research was to examine the relationship between perceived infectability, coronavirus anxiety, and psychological well-being. Correlational analysis confirmed that perceived infectability and coronavirus anxiety are both positively correlated and negatively related to psychological well-being. These findings are in line with the existing literature (Wang et al., 2019), indicating that high perceived infectability of the coronavirus leads to psychological distress (anxiety) among individuals.

Previous pandemic-related studies, dealing with the Ebola virus and severe acute respiratory syndrome (SARS), established similar phenomena that individuals who perceive the disease as life-threatening and contagious, experience more negative emotions such as anxiety (Wu et al., 2009). The plausible reasons could be the sudden and rapid transmission rate of the virus, since the transmission of COVID-19 is through the air (Zhong et al., 2020). This rapid transmission evokes anxiety among adults for a number of reasons, including high-risk perception, uncertainty of the future, and serious threats to both health and life itself.

Increased COVID-19-related anxiety (Ahuja et al., 2020) and perceived infectability are associated with a deterioration in psychological well-being (Ding et al., 2020). The plausible reasons for the increased anxiety could be deficient information about the virus, its rapid transmission, the non-availability of vaccine (to the youngsters and general public), elevated levels of perceived infectability, prompt changes such as self-isolation, social distancing, misleading information in the media, restrictions on travelling, and lockdowns. These factors further foster a disturbed mental state, prompting restlessness and uncertainty, and thus unfavorably influence the psychological well-being of the populace. Research on past pandemics also provides supportive evidence that other life-threatening illnesses like SARS also prompted a lower level of psychological well-being (Lau et al., 2008).

In addition, we explored the mediating role of Coronavirus anxiety in the relationship between perceived infectability and psychological well-being. Our findings emphasize that the addition of coronavirus anxiety as a mediator explains a further 14% variance. Constant worrying about health, and fear about the coronavirus and the future of the world, adds to coronavirus anxiety and thus hinders the daily functioning of the masses. People become more vulnerable to the perceived infectability of the virus, which is a real threat to their lives, overall, negatively affecting their psychological well-being.

Although there is no direct evidence concerning the mediating role of coronavirus anxiety in perceived infectability and psychological well-being, a recent study (Silva et al., 2021) suggested the mediating role of coronavirus anxiety in the relationship between mortality awareness and psychological well-being. Coronavirus anxiety is a variable which needs further exploration, as it is buffering many psychological issues and concerns including perceived infectability, which is a threat to individuals’ psychological well-being.

## Conclusion

Coronavirus anxiety significantly mediates the relationship between perceived infectability and psychological well-being. Future research needs to incorporate coronavirus anxiety as a factor, since the fear of the coronavirus, constant worrying about the future, and perceived infectability are potential risk factors for low psychological well-being. Furthermore, coronavirus anxiety management techniques need to be considered in providing community help programs and should also be included on coronavirus helplines. Interventions aimed at controlling the physiological aspects of the coronavirus outbreak should also include dealing with the psychological effects of the coronavirus.

## Limitations and Future Directions

The present study used a survey method based largely on network invitation rather than face-to-face random sampling, and thus the participants were required to be able to use or have knowledge about network tools. Therefore, one limitation of the study was that it did not include the sections of the population who cannot use network tools. Future research (post-pandemic) could avoid this issue by contacting individuals face to face and drawing a comparison between healthy and CO VID-19 victims. Lastly, the study configuration was cross-sectional; future research could use longitudinal study methods to grasp changes in psychological distress level and psychological well-being over the span of the COVID-19 pandemic.
